# Risk-Based Targeting of Animals for Ancillary Testing during a Bovine Tuberculosis Breakdown Is Associated with a Reduced Time to Test Failure: Indirect Evidence of *Mycobacterium bovis* Exposure?

**DOI:** 10.3390/pathogens13070606

**Published:** 2024-07-22

**Authors:** Andrew W. Byrne, Damien Barrett

**Affiliations:** 1 One-Health Scientific Support Unit, Department of Agriculture, Food and the Marine, Agriculture House, D02 WK12 Dublin, Ireland; 2 Ruminant Animal Health Division, Department of Agriculture, Food and the Marine, Backweston, Co. Kildare, W23 VW2C Celbridge, Ireland; damien.barrett@agriculture.gov.ie

**Keywords:** *Mycobacterium tuberculosis* complex, disease control, parametric survival analysis, Ireland

## Abstract

Bovine tuberculosis (bTB) continues to have significant economic and veterinary health impacts on cattle herds where the disease remains endemic. The continual tailoring of policies to address such maintenance requires an in-depth analysis of national data, underpinning new control strategies. In Ireland, when outbreaks occur, ancillary testing of herd mates deemed to be at the highest risk of exposure to reactors is undertaken using the interferon gamma (GIF) test. This highest risk cohort was hypothesised to be of a higher future risk despite this ancillary testing. We used a dataset from Ireland to model bovine test failure to the comparative tuberculin skin test using a survival analysis (observations: 39,248). Our primary exposure of interest was whether an animal that tested negative had a GIF test after the disclosure of infection within a herd during a bTB breakdown. There was evidence that animals with a negative GIF test during a breakdown had an increased risk of failing a test relative to other animals from the same herds without this exposure. The time to failure was 48.8% (95%CI: 38.3–57.5%) shorter for the exposed group relative to the unexposed group during a two-year follow-up period (2019–2022; time ratio: 0.51; 95%CI: 0.43–0.62; *p* < 0.001). The results from this study suggest that animals who were GIF-tested, having been deemed to have a higher risk of exposure, subsequently had shorter time-to-test failure periods. The absolute numbers of failure are small (only 2.5% of animals go on to fail during 2-year follow-up). Importantly, however, a high proportion of these high-risk herds included in the dataset failed at least one test at the follow-up (21/54 herds), impacting breakdown duration or recurrence. Such risk-informed targeting of animals could be utilised in future control policies, though further research is warranted.

## 1. Introduction

The eradication of bovine tuberculosis (bTB) remains a significant challenge in countries where *Mycobacterium bovis* is endemic in domestic hosts, especially where wildlife has been implicated [1]. In Ireland, despite the implementation of a long-term and intensive disease eradication programme, bTB has stubbornly persisted [2]. New policies adapted to the changing epidemiological situation are continually required [3]. Utilising routinely recorded national data is one means of effectively informing national policy formation in a timely and cost-effective manner [4,5]. 

In Ireland, interferon gamma (GIF) tests are used as ancillary tests during the management of bTB breakdowns, as disclosed by the presence of single intradermal comparative tuberculin test (SICTT) reactors or where post-mortem evidence of *M. bovis* infection has been identified in a herd [3,6]. GIF tests are known to exhibit higher test sensitivity than SCITT (e.g., 63–70% vs. 53–61%) but lower specificity (87–89% vs. 99.2–99.8%) under Irish conditions [7]—this pattern has been found across several studies, though the exact parameters can vary across studies, countries, and populations [8]. The GIF policy, first introduced on a phased basis in 2015, was used in larger herd breakdowns [3], and animals were targeted on the basis of their perceived level of exposure to reactors by the veterinarian managing the outbreak. All animals that were GIF positive were compulsorily removed in line with national and European legislation. However, animals that tested negative would revert to the same test policies as other herd mates that were not identified for GIF testing. We therefore hypothesised that GIF testing animals may have increased the risk of future test failure, relative to animals that were not GIF-tested, as they may have been exposed during the index cases within the breakdown. We assess the evidence for the elevated future risk of this cohort of animals by comparing their future test failure risk (time to test failure) relative to herd mates using survival models. 

## 2. Materials and Methods

We carried out a retrospective cohort study to assess the time-to-failure difference between animals that tested negative that were identified for ancillary testing during the management of a bTB breakdown using the results from a survival analysis acquired from national databases (AHCS and DAFM) from Ireland from 2019 to 2022. Details of the full dataset used in the present study can be found in Byrne et al. [5]. For our study population and eligibility criteria, we first identified cattle herds that experienced a breakdown between the 1 June 2019 and 31 December 2019, with the >1 standard SICTT reactor disclosed, and where GIF testing was deployed. Then, only herds which had four or more reactors during 2019 were included. This threshold was chosen to ensure that the breakdowns included in this study did not arise from the existence of a false positive breakdown. The probability of an average herd disclosing four false positive skin tests, at a specificity of 99.8% and a size of 149 animals, is 0.0003 [9]. As 58% of herds had 6 or more reactors during their outbreaks (Table 1), the probability reduced even further (<0.0001), indicating an extremely low likelihood that any of the herd breakdowns were due to false positive disclosure. Previous research has also shown that larger outbreaks are significantly associated with post-mortem evidence of infection (e.g., lesions) [10,11]. The study period was chosen to ensure that the uniform application of the policy and data recording within the national dataset used in this analysis were adhered to. We identified animals within breakdown herds that received a negative GIF test (“exposure”) and identified animals from the same herds (that would have also been tested within 30 days of the index GIF test) but never had a GIF test (our comparative group)—we called this binary predictor variable “GAMMA_EXP”. Only herds with a minimum of four or more “exposed” and “non-exposed” animals, respectively, were included in this study. Only animals that remained in their index herd throughout the study period were included to avoid the potential for differential risks associated with movement and differing exposure in the recipient (purchasing) farm.

Our outcome variable was the time to failure of the SICTT under standard interpretation (note, positive GIF tests were not events modelled during follow-up, as this would have biassed our study). Summary survival statistics were generated (incidence rate comparison) and graphically assessed using Kaplan–Meier curves. Initial differences amongst our “exposed” and comparative groups were assessed using log-rank tests. Univariable parametric survival models were fitted following Clegg and colleagues [12] with our primary predictor of interest, as well as the following potential confounding variables: age (quartiles), sex (m vs. f), breed (commercial dairy or other breeds (beef, dual, and rare breeds)), enterprise type (dairy or non-dairy [e.g., beef, suckler, and mixed] based on predominant cattle enterprise [13]) and herd size (categorised as small (mean: 58), medium (mean: 144), and large (mean: 323)), size of outbreak (minimum 4; categorised as ≤5, 6–10, >10), number of tests recorded, and two metrics of animal movement. A binary metric was used to indicate whether the birth herd was different than the testing herd (“moved since birth”) and whether the animal moved once or more than once since 2018. Descriptive statistics can be found in Table 1. Furthermore, additional models (Supplementary Material) were fitted including and excluding animals < 1 year old at entry, as it was hypothesised *a priori* (P. Breslin, pers. com.) that age could be a confounder given, for example, that the programme concentrated on cows and less so on heifers. Shared frailty was employed to account for the non-independence of observations from the same herd. 

Multivariable parametric models were fitted to the data [14], with comparisons being made amongst different distributions for the baseline hazard function (lognormal, exponential, Gompertz, log-logistic, Weibull, and generalised gamma). The choice of distribution was assessed by visualising the baseline hazard curve and the piece-wise exponential models and, primarily by, comparing models using Akaike’s and Bayesian Information Criteria (AIC and BIC, respectively) [15,16,17]. AIC and BIC were estimated sequentially, fitting models with each base hazard distribution and across two shared frailty distributions (gamma distribution and an inverse Gaussian distribution). Models with the lowest AIC and BIC were considered the preferred models. In terms of model building for the final predictor set, models were explored by fitting univariable survival models for each of the variables in Table 1, with only variables with unconditional associations at *p* < 0.2 being offered to the final model [15]. 

Final models reported time ratios (TRs) using the accelerated failure time (AFT) metric (non-proportional hazards model [15]; p. 507), whereby values < 1 represented shorter time to failure. Throughout the study, the STREG suite of tools within Stata 16 IC was used to model these data [18]. The alpha was set across models at *p* < 0.05. 

## 3. Results

The final dataset included 39,248 observations from 8753 animals in 54 large breakdown herds, of which 206 animals had a test failure during the follow-up (2.47%). There was an average of 156 (median: 109; IQR: 65–165) animals sampled per herd. The 206 animals with failures were from 21 herds (38.9% of all herds). Of these, 153 (74.3% of test failure animals; 1.83% of total animals) were GIF-tested during the index test across 18 herds (33.3% of herds). In comparison, 53 non-exposed animals failed a test during the follow-up within 15 herds (27.8% of herds). 

**Figure 1:**
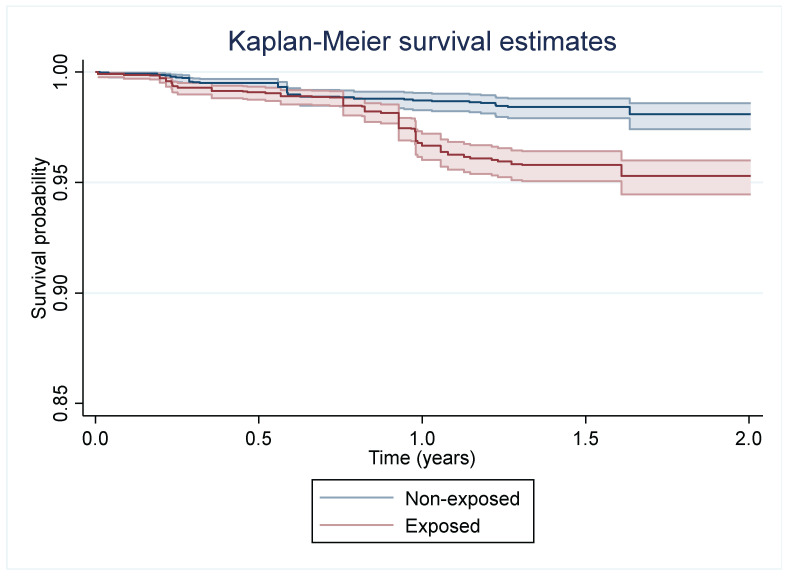
Kaplan–Meier curve of survival estimates over time since index test for animals who were tested using interferon gamma (GIF) test after breakdown, denoted as “exposed” group, relative to herd mates who were not GIF-tested, denoted as “non-exposed” group. Mean (median) time at risk: 1.18 (1.32) years.

The Kaplan–Meier curve for the exposure effect is presented in Figure 1 and illustrates a difference in survivorship over the follow-up period, particularly after day 300. The distribution with the lowest AIC and BIC was the log-logistic distribution with a gamma frailty, though there was little difference with the Weibull models (Δ < 2; Table S1). However, the exposure was found to be significantly associated (*p* < 0.001) with the outcome with similar parameter estimates irrespective of the underlying hazard function. Furthermore, univariable screening suggested associations between the time to failure and number of times tested (*p* < 0.001; Table 1). 

The final AFT multivariable model is presented in Table 2 and suggests that the mean survival time for exposed animals was 48.8% (95% CI: 38.3–57.5%)shorter relative to the non-exposed animals when controlling for the number of times tested. For each additional test an animal experienced, their median time to test failure increased by a factor of 1.46 (95%CI: 1.42–1.50). The frailty term was significant (Table 2; *p* < 0.001), indicating that there were significant clustering effects, such that the bTB test failure irrespective of exposure was higher in some herds relative to others. 

Despite age not being associated with a differential time to failure, an additional model was fitted to the data, but excluded all animals that were under 1 year old when recruited into the study (*n* = 7099 animals, 15% reduction from the full model). This model suggested that the time to failure was significantly shorter for the GAMMA_EXP animals relative to the comparative animals, with a 29.1% shorter survival time (95%CI: 17.1–39.3%; Supplementary Material Table S2).

## 4. Discussion and Conclusions

During this study, it was found that the negative GIF-tested animals had a significantly shorter time to failure during the follow-up period relative to the animals from the same herds who were not selected for GIF testing. Given that GIF testing was targeted at the perceived highest-risk cohorts within breakdown herds by the attending veterinarian, we suggest that these data support the hypothesis that these animals were indeed at a higher risk of exposure during breakdowns (and therefore at a higher risk of future test failure). Furthermore, this interpretation of the data suggests that a cohort of these GIF-tested animals were false negative cases to both GIF and SICTT around the index test time. Importantly, this is despite the increased sensitivity of the GIF test relative to the SICTT [7,8,19]. Having the GIF test as an ancillary test after the SICTT, in effect, parallel testing, should have helped increase the overall sensitivity of the test regime (though at the potential cost of specificity) [19]. In the absence of removing GIF positive animals, the effect size of the exposure metric would likely have been significantly larger given previous evidence [20,21,22]. For example, Gormley and colleagues [22] found that skin test negative–GIF positive animals had 7–9 times greater odds of test failure over a 1.5 year follow-up period relative to SICTT negative– GIF negative herd mates.

An alternative interpretation of the data was that the GIF-tested animals were more stringently tested during the follow-up period and were therefore at higher levels of surveillance scrutiny. While this hypothesis is possible, a model controlling for the number of tests an animal received over the follow-up period revealed that GAMMA-EXP remained significant. Furthermore, there is no direct provision for this type of policy within the bTB programme in Ireland [23] as GIF negative animals return to the same testing regime as their herd mates who were not GIF-tested. The Kaplan–Meier curve suggested increased risk, particularly after day 300, which may be related to the scheduling of tests for these high-risk herds. In Ireland, high-risk herd testing involves reactor retests every 60 days (until two clear herd tests are found) and then an additional TB check test protocol with higher frequency testing for up to 20 months after the original breakdown commenced [24]. This may indicate that other factors may be at play, potentially including delays from exposure to being detectable (and detected) with tuberculin tests or potentially secondary transmissions occurring during the breakdown period within the exposed cohorts [25,26]. 

Several independent variables were tested by fitting univariable models (Table 1) based on prior experience and published studies in the literature (e.g., dairy animals having a higher future risk [20]); however, none were found to be significantly associated with time to failure in the present study. This may be due to the study size and the power to detect effects or other attributes related to this specific study. Indeed, even though the number of observations was high, the power of a survival analysis is determined by the number of failures (events) [27], which was modest in this study. The final model in the present study had a significant frailty term, indicating that the test failure risk also clustered amongst certain higher-risk herds. This reflects the chronic nature of the bTB epidemiology in Ireland, where recrudescence occurs from within-herd maintenance due to failure to clear all infections but also from external disease incursion risk, including wildlife [26]. 

An important point to note with the findings from this study is that, for animals from breakdown herds that test negative for either GIF or the SICCT, the absolute risk of future test failure was very small. This was also highlighted by other recent research [5,6]. For example, Madden and colleagues [6] found that only 3.37% (926/27,518) of animals tested for GIF in Ireland went on to fail a follow-up SICTT (reactors) over a two-year period. In another smaller study in a population where severe breakdowns occurred, 7.23% (35/484) of animals failed an SCITT at a follow-up of two years [28]. In the present study, 97.5% of the included animals did not fail an SICTT at the follow-up, showing that the vast majority of animals do not fail standard diagnostic tests even within the exposure group. Despite these low numbers, the consequence in terms of future breakdowns, recrudescence, and spread is high, and it is possibly an underestimate given the sensitivity of routine surveillance, at the animal level, that could lead to undetected infections [29]. The herd-level impact was demonstrated by the fact that a high proportion of the herds included in this study had a recorded test failure during the follow-up (38.9% of herds disclosed a failure during the follow-up). This would lead to significant increases in the duration of the breakdown, as the disclosure of animals that tested positive would lead to additional 60-day testing or recrudescence if the failure occurred after restrictions from the index breakdown were lifted. Both outcomes would contribute on-going costs to stakeholders, including farmers and the state [3].

There were several limitations in this study. Firstly, this was a retrospective, cohort, observational study, and therefore, causality cannot be absolutely established. There is a risk that some of the variation in future risk in GIF-tested animals could be attributed to the skills of the veterinarian undertaking the epidemiological investigation to identify and test the exposed cohort, hence contributing to the significant clustering effects at the herd level. However, there was a protocol for the programme to test animals with direct contact with reactor animals. Future studies could investigate whether there are “investigation-level” factors that could hone the epidemiological investigations to improve the identification of the highest-risk animals. 

Previous research from Ireland and elsewhere has highlighted the emerging need to gain greater insights into routinely collected data and the need for more real-time analytic products for disease control stakeholders [4,5]. The present study has added to this evidence-informed policy-making process in Ireland, but, given the uncertainties attributable to the mechanisms underlying the risk difference, additional research is warranted. 

## Figures and Tables

**Table 1:** Descriptive statistics and univariable associations (parametric survival model with log-logistic hazard distribution and a gamma frailty) with independent variables assessed during model building to assess their effects on relationship between GIF exposure and time-to-test failure risk. TR: time ratio from AFT model.

Animal Level						
		*n*	%/Median	IQR	TR	*p*
**Age (years)**	Median (IQR)	8753	3.81	2.23–5.97	0.988	0.669
**Sex**	Female	7059	84.48%		ref	
	Male	1297	15.52%		1.093	0.746
**Breed**	Commercial dairy	6026	68.85%		ref	
	Other	2727	31.15%		0.802	0.390
**Moved since birth**	No	6890	78.72%		ref	
	Yes	1863	21.28%		0.799	0.409
**Moves since 2018**	0	7467	85.31%		ref	
	1	1060	12.11%		0.804	0.505
	>1	220	2.51%		0.478	0.217
**No. tests**	Median (IQR)	8753	5	3–7	1.724	<0.001
**Total N**		8753				
**Herd level**						
**Herd type**	Dairy	32	59.26%		ref	
	Non-dairy	22	40.74%		1.881	0.315
**Herd size**	Small	18	61	44–74	ref	
	Medium	18	149	117–164	2.096	0.338
	Large	18	245	208–324	1.289	0.739
**Outbreak size**	4–5 reactors	22	40.74%		ref	
	6–10 reactors	17	31.48%		1.252	0.763
	11 or more reactors	15	27.78%		1.519	0.592
**Total N**		54				

**Table 2:** Multivariable parametric survival model for time to failure for interferon gamma-tested animals after a bTB breakdown. TR: Time ratio from AFT model; hazard distribution: log-logistic distribution; frailty: gamma.

	TR	Std. Err.	z	*p*	Lower 95% CI	Upper 95% CI
**Non-exposed**	ref					
**Exposed**	0.512	0.049	−7.050	0.000	0.425	0.617
**no. times tested**	1.457	0.020	26.780	0.000	1.417	1.497
**Constant**	0.498	0.129	−2.700	0.007	0.300	0.827
**ln(theta)**	2.446	0.253	9.690	0.000	1.951	2.941
Theta *	11.543	2.915			7.036	18.936

* Likelihood-ratio test of theta = 0: *p* < 0.001; indicates whether the variance in frailties across herds are significantly heterogeneous.

## Data Availability

The datasets presented in this article are not readily available because of national legislation and privacy restrictions. Requests to access the datasets should be directed to opendata@agriculture.gov.ie.

## Supplementary Materials

The following supporting information can be downloaded at: https://www.mdpi.com/article/10.3390/pathogens13070606/s1, Table S1: Parameter estimates for exposure against time to failure fitted with a gamma frailty and several hazard distributions for parametric survival models. Estimates are time-ratios for all accelerated failure time models, with the exception of the Gompertz model which is a proportional hazard model; Table S2: Parametric survival model for time-to-failure for animals exposed to GIF testing after a TB breakdown over a follow-up period of 2 years, excluding all animals recruited < 1year of age. Distribution: Log-logistic; Frailty: gamma.
