# Supplementary material for: Risk-Based Targeting of Animals for Ancillary Testing during a Bovine Tuberculosis Breakdown Is Associated with a Reduced Time to Test Failure: Indirect Evidence of Mycobacterium bovis Exposure?
Source: Pathogens. 2024 Jul 22;13(7):606. doi: 10.3390/pathogens13070606 (PMC11280051; doi:10.3390/pathogens13070606)
Supplement: Supplementary file 1 [file pathogens-13-00606-s001.zip › pathogens-3097810-supplementary.pdf]

Table S1: Parameter estimates for exposure against time to failure fitted with a gamma frailty and several hazard distributions for parametric survival models. Estimates are time-ratios for all accelerated failure time models, with the exception of the Gompertz model which is a proportional hazard model.

Hazard distribution	Rank	GAMMA_EXP	GAMMA_EXP P-value	No. tests	No. tests P-value	Constant	df	AIC	BIC
**log-log**	**1**	0.512	<0.001	1.457	<0.001	0.498	5	1417	1460
**Weibull**	**2**	0.449	<0.001	1.458	<0.001	0.612	5	1419	1461
**log-normal**	**3**	0.541	<0.001	1.491	<0.001	1.265	5	1456	1499
**Gompertz**	**4**	5.817*	<0.001	0.424*	<0.001	0.230	5	1473	1516
**exponential**	**5**	0.275	<0.001	1.746	<0.001	3.274	4	1615	1649
**g-gamma^**	**6**	0.349	0.002	1.391	0.002	7.133	5	2044	2087

* Hazard ratio; ^ Generalised gamma; no shared frailty, but fitted with vce(cluster) option where SE are adjusted for intragroup correlation.

Table S2: Parametric survival model for time-to-failure for animals exposed to GIF testing after a TB breakdown over a follow-up period of 2 years, excluding all animals recruited <1year of age. Distribution: Log-logistic; Frailty: gamma.

	Time Ratio	Std. Err.	z	P>z	lower 95% CI	Upper 95% CI
**Non-exposed**						
**Exposed**	0.709	0.056	-4.320	0.000	0.607	0.829
**no. times tested**	1.451	0.019	27.790	0.000	1.413	1.489
**Constant**	0.403	0.066	-5.530	0.000	0.292	0.556
**ln(theta)**	2.413	0.253	9.530	0.000	1.917	2.909
**gamma**	0.311	0.019			0.277	0.349
**Theta***	11.169	2.828			6.799	18.347
